# ReliefF-Based EEG Sensor Selection Methods for Emotion Recognition

**DOI:** 10.3390/s16101558

**Published:** 2016-09-22

**Authors:** Jianhai Zhang, Ming Chen, Shaokai Zhao, Sanqing Hu, Zhiguo Shi, Yu Cao

**Affiliations:** 1 College of Computer Science, Hangzhou Dianzi University, Hangzhou 310018, China; jhzhang@hdu.edu.cn (J.Z.); cming163@163.com (M.C.); lnkzsk@126.com (S.Z.); 2 Department of Information Science and Electronic Engineering, Zhejiang University, Hangzhou 310012, China; shizg@zju.edu.cn; 3 Department of Computer Science, The University of Massachusetts Lowell, Lowell, MA 01854, USA; ycao@cs.uml.edu

**Keywords:** ReliefF, EEG, sensor selection, emotion recognition

## Abstract

Electroencephalogram (EEG) signals recorded from sensor electrodes on the scalp can directly detect the brain dynamics in response to different emotional states. Emotion recognition from EEG signals has attracted broad attention, partly due to the rapid development of wearable computing and the needs of a more immersive human-computer interface (HCI) environment. To improve the recognition performance, multi-channel EEG signals are usually used. A large set of EEG sensor channels will add to the computational complexity and cause users inconvenience. ReliefF-based channel selection methods were systematically investigated for EEG-based emotion recognition on a database for emotion analysis using physiological signals (DEAP). Three strategies were employed to select the best channels in classifying four emotional states (joy, fear, sadness and relaxation). Furthermore, support vector machine (SVM) was used as a classifier to validate the performance of the channel selection results. The experimental results showed the effectiveness of our methods and the comparison with the similar strategies, based on the F-score, was given. Strategies to evaluate a channel as a unity gave better performance in channel reduction with an acceptable loss of accuracy. In the third strategy, after adjusting channels’ weights according to their contribution to the classification accuracy, the number of channels was reduced to eight with a slight loss of accuracy (58.51% ± 10.05% versus the best classification accuracy 59.13% ± 11.00% using 19 channels). In addition, the study of selecting subject-independent channels, related to emotion processing, was also implemented. The sensors, selected subject-independently from frontal, parietal lobes, have been identified to provide more discriminative information associated with emotion processing, and are distributed symmetrically over the scalp, which is consistent with the existing literature. The results will make a contribution to the realization of a practical EEG-based emotion recognition system.

## 1. Introduction

The effort to integrate emotions into a human-computer interaction (HCI) system has attracted broad attention for a long time and has resulted in a new research field of affective computing [1]. Affective computing aims to give computers the ability to perceive human emotions and then make proper decisions to respond. Affective computing has been becoming a worldwide research hotspot in recent years, partly due to the quick development of wearable computing and the urgent needs of a more immersive HCI environment. The key element in affective computing is to detect human emotions accurately in real time by employing pattern recognition and machine learning techniques. Although numerous studies have been performed, emotion recognition is still an extremely challenging task, especially for the purpose of practical usage.

A variety of methods for detecting human emotion have been proposed using physical or physiological measurements in the past few decades, such as facial expression, speech, body gesture, electrocardiograph (ECG), skin conductance (SC), electromyogram (EMG), respiration, pulse, etc. [2,3,4]. Although some encouraging results have been achieved, these measurements are an indirect mapping of human emotions. Recently, electroencephalogram (EEG)-based emotion recognition has received increasing attention. In comparison with the aforementioned measurements, EEG technique can directly detect the brain’s dynamics in response to different emotional states, so it is expected to provide more objective and comprehensive information for emotion recognition. Furthermore, with the advantages of non-invasion, low-cost, portability and high temporal resolution, it is possible to use an EEG-based emotion recognition system for real-world applications.

Although the excellent temporal resolution makes EEG a primary option for a real-time emotion recognition system, the poor spatial resolution of EEG presents a big challenge for the task. To overcome this problem, multi-channel EEG sensor signals are used, often acquired from 32, 64 or even more sensor electrodes distributed on the whole scalp according to the International 10–20 system. It is supposed that the wealthy sensors can provide more discriminative information of different emotional states and improve the recognition performance. Unfortunately, the rule of “the more the better” is not always right in this situation. In fact, additional EEG channels usually include noisy and redundant channels, which would instead deteriorate the recognition performance [5,6,7]. Moreover, a large set of sensor channels will increase the computational complexity and cause users inconvenience. Two solutions have been used to tackle this problem, consisting of feature selection and EEG sensor channel selection. So far, related studies are mainly focused on selecting the least useful features to achieve the best performance. Even if the reduction of features usually leads to a reduction of channels involved, it cannot guarantee the selected channels are optimum. In addition, reduction in channels is more important than features reduction from a practical point of view. Fewer channels can not only reduce the computational complexity and improve the performance, but also facilitate the practical usage. It is therefore necessary to investigate the problem of sensor channel selection directly.

Various strategies have been used for sensor selection in research of EEG-based emotion recognition. In some studies, sensors were selected according to the expert’s experience. Bos [8], Zhang and Lee [9] and Schmidt et al. [10] selected sensors for classifying two emotional states according to the well-known frontal asymmetry of the alpha power for valence. A set of symmetric sensor electrode pairs were used to recognize more emotional states by Valenzi et al. [11] and Lin et al. [12], employing hemisphere asymmetrical characteristics of emotion processing in the human brain. These works mainly focused on few specific features at specific scalp locations. However, the state-of-the-art findings in neuroscience recommend investigating the correspondence between emotional states and whole brain regions [13]. Therefore, finding the optimal subset of sensor channels from the full set automatically has attracted increasing attention recently. Two main approaches have been used in sensor selection algorithms for EEG signal processing [14]. One of them was directly based on a feature selection algorithm. Firstly, a specific feature selection method was used to find the top-N features according to some criteria, and then the electrodes containing these selected features were chosen. Wang et al. [15] applied minimum redundancy maximum relevance (mRMR) feature selection method to find the best electrode positions of TOP-30 features among all 64 electrodes. The average selected sensor number was 25 with five subjects. Employing a feature selection method based on the F-score index, Lin et al. [12] reduced the number of required electrodes from 24 to 18 at the expense of a slight decrease in classification accuracy. The results of this kind of approach showed its effectiveness, but not its optimum for channel selection in most cases. Another kind of approach for sensor channel selection in literature adopted a classification algorithm to evaluate the candidate sensor subsets generated by a search algorithm. In Jatupaiboon et al. [16], support vector machine (SVM) was used to evaluate a different electrode montage from seven pairs (14 channels) for classifying positive and negative emotions. Finally, the number of electrodes was reduced from seven pairs to five pairs almost without any loss of accuracy. With sufficient searching, optimal results could be obtained with this kind of approach, but it depends on the specific classification algorithm and is computationally expensive.

In the present study, we systematically investigated a channel selection scheme based on ReliefF [17] in EEG-based emotion recognition. The ReliefF algorithm is a widely used feature selection method. Different to the aforementioned feature-selection-based channel selection method, after obtaining the weights of all features by ReliefF, two strategies were used to quantify the importance of every sensor channel as a unity by considering the weights of all features belonging to this channel. Then the channels were directly selected according to their quantified importance in emotion recognition. SVM as classifier was used to evaluate the effectiveness and performance of our methods. Furthermore, similar strategies using the F-score as a feature selection method were also investigated and compared.

The rest of this paper is structured as follows. Section 2 presents the material and methods, including the description of the database for emotion analysis using physiological signals (DEAP), feature extraction, channel selection based on an ReliefF algorithm, and the an SVM classifier. Section 3 is dedicated to the obtained results. The proposed methods are assessed in the task of classifying four emotional states with SVM and compared with the F-score methods. The problem of selecting subject-independent channels for emotion recognition is also investigated. Related discussion is given in Section 4. Finally, we conclude the proposed approach in Section 5.

## 2. Materials and Methods

### 2.1. DEAP Database

This study was performed on the publicly available database DEAP [18], which consists of a multimodal dataset for the analysis of human emotional states. A total of 32 EEG channels and eight peripheral physiological signals of 32 subjects (aged between 19 and 37) were recorded whilst watching music videos. The 40 one-minute long videos were carefully selected to elicit different emotional states according to the dimensional, valence-arousal, emotion model. The valence-arousal emotion model, first proposed by Russell [19], places each emotional state on a two-dimensional scale. The first dimension represents valence, ranged from negative to positive, and the second dimension is arousal, ranged from calm to exciting. In DEAP, each video clip is rated from 1 to 9 for arousal and valence by each subject after the viewing, and the discrete rating value can be used as a classification label in emotion recognition [20,21,22].

In this study, for simplicity, the preprocessed DEAP dataset in MATLAB format was used to test our channel selection algorithms for classifying four emotional states (joy: valence ≥5, arousal ≥5; fear: valence <5, arousal ≥5; sadness: valence <5, arousal <5; relaxation: valence ≥5, arousal <5). In the preprocessing procedure, the sampling rate of the EEG signal was down sampled from 512 Hz to 128 Hz and a band pass frequency filter from 4.0–45.0 Hz was applied. In addition, electrooculography (EOG) artifacts have been removed from the EEG signal. For each subject, there are 40 one-minute trials which were divided into four categories labeled as joy, fear, sadness or relaxation, separately. In our experiment, about half of the trials for each category were randomly selected for channel selection (channel-selection dataset), and the rest were used to test the performance of the channel selection results (performance-validation dataset). To make sure that there are relatively enough data in every category for the channel selection and the test, only the subjects in their dataset, with a number of trials for every category that is no less than 5, were considered. Thus, sixteen subjects (1, 2, 5, 7–11, 14–19, 22, 25) were chosen. Candra et al. [23] reported that the effective window size for arousal and valence recognition using the DEAP database was between 3–10 and 3–12 seconds respectively. So, each 60-s trial was segmented into 15 4-s samples with non-overlapping for the purpose of increasing the number of samples. Finally, we got a total of 600 (40 trials × 15 samples) samples for each subject. All the samples derived from the same trial share the same category label.

### 2.2. Feature Extraction

Different types of features have been used in EEG-based emotion recognition, including time, frequency or time-frequency domain features [24]. However, power features from different frequency bands are still the most popular in the context of emotion recognition. In this work, a series of band pass filters were used to translate the raw EEG data from 32 channels of each sample to the ta (4–8 Hz), alpha (8–13 Hz), beta (13–30 Hz) and gamma (30–45 Hz). Then, the power of every specific frequency band was calculated using a 512-point fast Fourier transform (FFT), so 128 (32 channels × four features) features were obtained for each sample.

The power of a specific frequency band corresponding to channel *T* in sample *R* is calculated as

(1) $$bp_{RT}=\frac{1}{N}{\sum_{k=1}^{N}\left|X_{N}^{RT}(k)\right|}^{2},$$

where, $X_{N}^{RT}(k)$ is the FFT of the EEG signals for channel *T* in sample *R*, *N* is the length of FFT and equals the sample length 512 (4 s). Z-score normalization was adopted for each feature [25]. For a feature *f_R_* belonging to sample *R*, the normalized value was computed by

(2) $$f_{R}^{norm}=\frac{f_{R}-\mu_{f}}{\sigma_{f}},$$

where, *µ_f_* and *σ_f_* are, respectively, the mean and the standard deviation of feature *f* over all samples.

### 2.3. Channel Selection Based on ReliefF

ReliefF is a widely used feature selection method in classification problems, due to its effectiveness and simplicity of computation. A key idea of ReliefF is to evaluate the quality of features according to their abilities to discriminate among samples that are near to each other. The ability is quantified as a weight of every feature. Channel selection can be performed based on the results of ReliefF feature selection, just as the typical feature-selection-based channel selection method aforementioned.

In our work, 128 features were used to discriminate four emotional states (joy, fear, sadness, relaxation) with the channel-selection dataset—about 300 samples of every subject. Firstly, a ReliefF algorithm was used to rank all the 128 features according to their weights. At the beginning, all the weights for 128 features were set to zero. For each sample *R_i_*, the *k* nearest neighbors from the same class of *R_i_* (nearest hits *H_j_*) and *k* nearest neighbors from each of the different classes (nearest misses *M_j_*(*C*)) were found in terms of the features’ Euclidean distance between two samples. Then, the weights *W*(*F*) for all features were updated according to Equation (3). The above process was repeated until all the samples in the channel-selection dataset had been chosen, and the final *W*(*F*) was our estimations of the qualities of 128 features. The whole process is implemented with the following steps:

1. set all the weights of 128 features to zero, $W(F)=W[f_{1},f_{2},\ldots ,f_{128}]:=0.0$;
2. for *i* = 1 to the number of samples in the channel-selection dataset do
3. select a sample *R_i_*;
4. find *k* nearest hits $H_{j}(j=1,2,\ldots ,k)$;
5. for each class *C* ≠ class(*R_i_*) do
6. find k nearest misses *M_j_*(*C*)(*j* = 1, 2, …, *k*) from class *C*;
7. end;
8. for *l* = 1 to 128 do
  (3) $$\begin{array}{l} W(f_{l})=W(f_{l})-\sum_{j=1}^{k}diff(f_{l},R_{i},H_{j})/(m\times k)+\\ \sum_{C\neq class(R_{i})}\left[\frac{P(C)}{1-P(class(R_{i}))}\sum_{j=1}^{k}diff(f_{l},R_{i},M_{j}(C))\right]/(m\times k) \end{array};$$
9. end;
10. end;

where, the nearby neighbor’s number *k* is 10, which is safe for most purposes [17] and the prior probability of class *C* is *P(C)* (estimated by the channel-selection sets). Function $diff(f,R_{1},R_{2})$ calculates the difference between values of the feature *f* for two samples *R_1_* and *R_2_*, which is defined as:

(4) $$diff(f,R_{1},R_{2})=\frac{\left|value(f,R_{1})-value(f,R_{2})\right|}{\text{max}(f)-\text{min}(f)}$$

where, *value*(*f*,*R*) is the value of feature *f* in sample *R*, max(*f*) and min(*f*) are respectively the maximum and minimum of the feature *f* over all samples.

Through the above described process, it can be easily understood that a large weight means the feature is important to discriminate between samples and a small one means that it is less important. Therefore, all features can be ranked in terms of their weights. While selecting channels, the top-N features were chosen according to the rank of every feature, and then the channels containing these features were selected. Although the reduction of features usually led to a reduction of channels involved, the actual effect was not obvious [12]. 

However, the ultimate goal of sensor channel selection is to achieve the best accuracy for classification by using the least number of sensors. From this perspective, a natural strategy is evaluating the importance of channels directly for classification, treating a channel rather than a feature as a unit. If we accept that the weight of a feature obtained from ReliefF reflects its capability to discriminate different classes, we can use the weights of all features belonging to a channel to estimate the channel’s contribution to class discriminability.

We defined the mean of the weights of all features belonging to a channel as this channel’s weight. In our work, 32 channels were used and each channel contained four features. The weight of channel *T* is computed as

(5) $$W(T)=\frac{1}{N}\sum_{i=1}^{N}W(f_{i}),$$

where, *W*(*f_i_*) is the weight of the *i*-th feature (*f_i_*) belonging to channel *T*, and *N* is the number of features of channel *T*. Then, channels can be ranked according to their weights, and the best channels for the classification task can be easily selected. This method was denoted as mean-ReliefF-channel-selection (MRCS).

**Figure 1:**
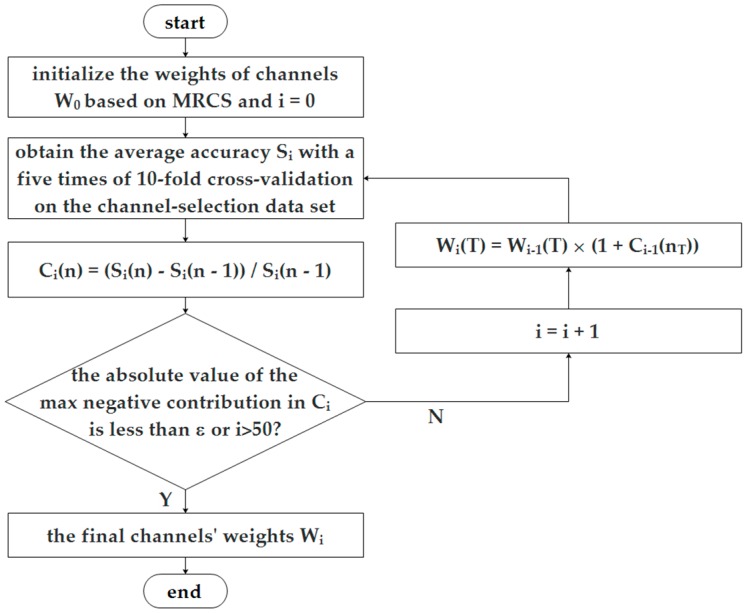
The update process of channels’ weights considering their contribution to classification accuracy.

Now, we can select channels, independent of a classifier, for a further classification task. It is perhaps effective for different kinds of classifiers, but it cannot be guaranteed to be optimal for a specific classifier. As mentioned in Section 1, a search algorithm can find the optimal channel set for a specific classifier, but it is computationally expensive. We propose a strategy to iteratively adjust the weights of channels according to their contribution to classification accuracy for a specific classifier, as in Figure 1. At the beginning, the weight of every channel is initialized to the mean of the ReliefF weights of all features belonging to this channel as *W*_0_(*T*). Then, on each subject’s channel-selection dataset, the average accuracy over a varying number of channels is obtained using a specific classifier by adding the channels one by one according to their weights, labeled as S_0_(*n*) (the average classification accuracy, when top-n channels are used, *n* = 1 to 32). Then the contribution of the top *n*-th channel in the *i*-th iteration can be computed as

(6) $$C_{i}(n)=\frac{S_{i}(n)-S_{i}(n-1)}{S_{i}(n-1)},$$

this contribution value of a channel could be positive or negative. Then, the weight of channel *T* is updated as

(7) $$W_{i}(T)=W_{i-1}(T)\times (1+C_{i-1}(n_{T})),$$

where, *n_T_* are the rank of channel *T* in all 32 channels. It means that the weight of a channel increases when its contribution is positive and decreases in the reverse case. The above process is repeated until the absolute value of the max negative contribution of all channels is less than ε or iterates 50 times. In this paper, ε equals 0.01. Then, the importance of channels will be evaluated and selected according to the final weights *W*. The purpose of this method is to further optimize the channel selection performance against a specific classifier based on MRCS, denoted as X-MRCS (X can be replaced with classifier name, such as SVM-MRCS).

### 2.4. Classifiers to Evaluate the Performance

To validate and compare the effectiveness of the channel selection results of our methods, SVM was employed as classifiers to recognize four emotional states using the selected sensors on a performance-validation dataset. The central idea of SVM is to separate data from two classes by finding a hyperplane with the largest possible margin. The SVM classifier used in our work was implemented by LIBSVM (a library for support vector machine developed by Chang and Lin [26]) with the radial basis function (RBF) kernel. The one-versus-one strategy for multi classification problems was applied. To reduce the computational cost, we just let the penalty parameter be *C* and the kernel parameter be *γ* as default. For each subject, the average classification accuracy was computed using a scheme of five 10-fold cross-validations to increase the reliability of our results. In a 10-fold cross validation, all the validation samples are divided into 10 subsets. Nine subsets are used for training, and the remaining one is used for testing. This process was repeated five times with different subset splits. The classification accuracy was evaluated by the ratio of a correctly classified number of samples and the total number of samples. Then, the average classification accuracy curve, over a varying number of selected channels across all 16 subjects, was given to illustrate and compare the performance of the channel selection methods. Furthermore, the related results were statistically evaluated by a paired t-test method to further support and validate our conclusion.

## 3. Results

In this section, we evaluated our ReliefF-based sensor selection algorithms in the task of classifying four emotional states on a DEAP dataset, with SVM as the classifier. Just as mentioned in Section 2, 16 subjects (1, 2, 5, 7–11, 14–19, 22, 25) among 32 were chosen in our experiment. In addition, all the trials for each subject were divided into two subsets according to the aforementioned rules. About half of the trials were used as the first subset for channel selection, and the rest were used to validate the performance of the channel selection results. Three ReliefF-based sensor selection strategies were employed. In the first strategy, after obtaining the weights of all features, sensor channels containing the top-N features were selected. For the second method MRCS, the mean of the weights of all features belonging to a channel, was used as this channel’s weight and all channels were ranked according to their weights for selection. In SVM-MRCS, channels were selected considering their weights and their contribution to classification accuracy against the classifier SVM. After obtaining every channel’s weight for each subject on the channel-selection dataset, the classification accuracy of selected top-N channels, with five 10-fold cross-validations, on the performance-validation dataset was achieved. Then, the average classification accuracy across 16 subjects over a varying number of channels was given to show the effectiveness of our methods. For comparison, similar strategies were also applied to the F-score algorithm. Finally, the subject-independent channel selection problem was investigated.

### 3.1. Subject-Dependent Channel Selection

**Figure 2:**
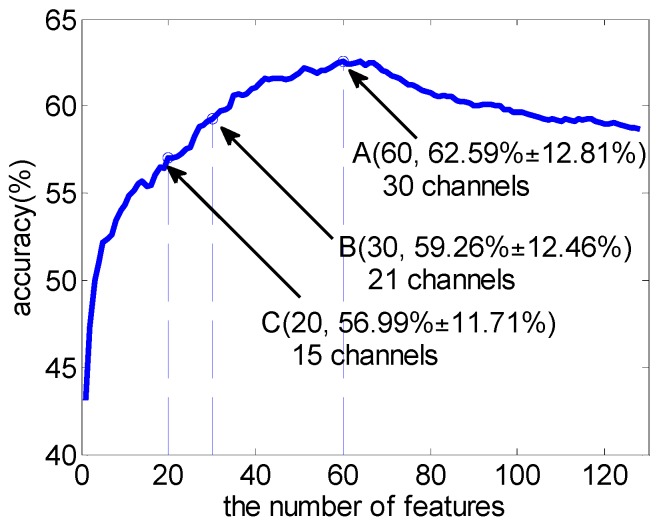
Average classification accuracy over varying number of features across 16 subjects with SVM. Point A achieves the best classification accuracy.

Firstly, the subject-dependent ReliefF weights of 128 features were computed on the channel-selection dataset. Then SVM was used to validate the feature selection results on the performance-validation dataset. Figure 2 shows the average classification accuracy using SVM over a varying number of features across 16 subjects. The classification accuracy curve was obtained by adding the features one by one, according to their ReliefF weight. Features with larger weight will be added first. From Figure 2, it shows that the best classification performance was achieved at a certain point of the feature number (point A, 62.59% ± 12.81% accuracy using 60 features and 30 channels involved), and then degraded with the increase of the feature number, just as we had expected. The results show the effectiveness of ReliefF as a feature selection algorithm in emotion recognition. However, though the number of features was reduced dramatically, the reduction of the number of channels involved was not obvious (at point C, 20 features still need 15 channels). Table 1 demonstrates the average number of channels involved and the average classification accuracy when using top-N features across 16 subjects. In addition, Table 2 presents the average proportion of each class of features among the top-N features. It can be found that the high frequency bands (beta, gamma) play a more important role in emotion processing. Similar results have also been reported in other literatures [10,16,27], which prove the significance of ReliefF weight to some extent.

**Figure 3:**
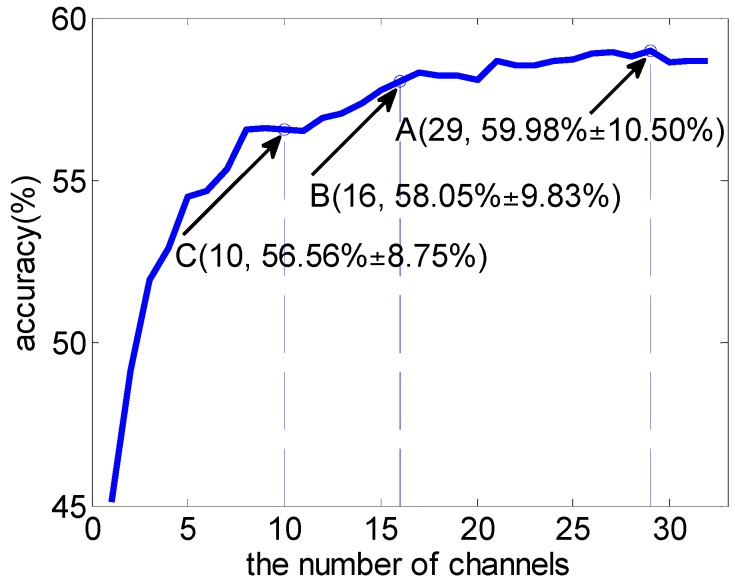
Average classification accuracy over varying number of channels using SVM based on the MRCS method. Point A achieves the best classification accuracy.

With all the features’ weights computed above, we could evaluate the importance of channels in the emotion classification task directly by means of the weights of all features belonging to a channel, according to the MRCS method. Figure 3 shows the average classification accuracy using SVM over a varying number of channels across 16 subjects. Channels were added one by one according to their weights (Equation (5)). Different to the first feature-selection-based channel selection method, all the features of the selected channels would be fed to a classifier.

In Figure 3, employing the MRCS method, the best classification performance was achieved by using the top 29 channels. It is worth noting that, when the number of channels was reduced from 29 (point A) to 16 (point B), the average accuracy only slightly reduced. It shows good performance in channel reduction. Comparing point C in Figure 2 and Figure 3, there was a similar average classification accuracy (56.99% ± 11.71% vs. 56.56% ± 8.75%), but the number of channels involved reduced from 15 to 10.

**Figure 4:**
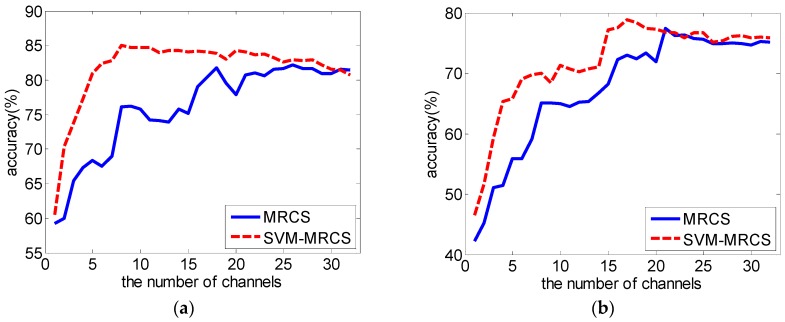
SVM average classification accuracy over a varying number of channels for subject 7 based on MRCS (blue solid line) and SVM-MRCS methods (red hashed line). (**a**) adjustment on the channel-selection dataset; (**b**) evaluation on the performance-validation dataset.

The above two channel selection methods were independent of the classifier. Can we improve the performance by considering channels’ contribution to classification accuracy, when using a specific classifier, following the process of Figure 1? Figure 4 takes subject 7 as an example to illustrate the result of the adjustment with SVM as the classifier (SVM-MRCS method). All the adjustment was done on the channel-selection dataset (Figure 4a) according to the procedure in Figure 1, and the adjustment result was evaluated on the performance-validation dataset with the same classifier (Figure 4b). All the average classification accuracy was computed using a scheme of five 10-fold cross-validations. It can be seen from Figure 4b that the performance was improved by adjusting channels’ weights according to their contribution to classification accuracy (paired *t*-test, significant difference *p* < 0.05 when the number of selected top-N channels is less than 20).

**Figure 5:**
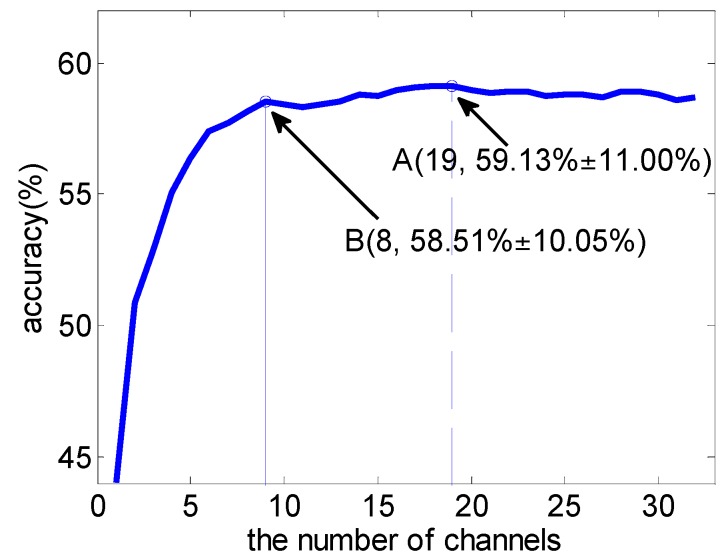
Average classification accuracy over a varying number of channels using SVM based on the SVM-MRCS method. Point A achieves the best classification accuracy.

**Figure 6:**
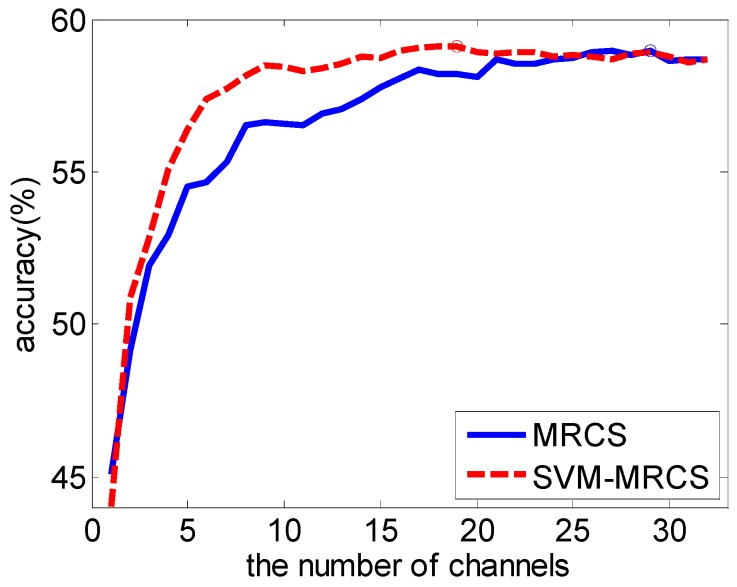
Average performance comparison across 16 subjects between MRCS and SVM-MRCS using SVM.

The average classification accuracy over a varying number of channels based on the SVM-MRCS method across 16 subjects was shown in Figure 5. It shows that the best classification accuracy can be achieved by using the top 19 channels (point A). More importantly, the accuracy curve becomes almost stable when the top eight channels (point B) were used (with a slight reduction of 1% compared to point A using 19 channels). Figure 6 and Table 3 show the comparison of the average performance across 16 subjects between MRCS and SVM-MRCS using SVM. From the comparison, SVM-MRCS demonstrates better performance than MRCS in channel selection (paired *t*-test, with significant difference *p* < 0.05 when the number of selected channels is between 6 and 14). Comparing these three channel selection methods, although the best classification performance of the latter two methods is slightly reduced (about 3%), the number of channels was more dramatically reduced than the first one. Especially, while a lower number of channels (less than 10 for example) is needed, MRCS and SVM-MRCS have an obvious advantage. However, because a specific classifier was employed in the SVM-MRCS method, computational complexity increased and the selection result perhaps was not the optimum for other classifiers.

### 3.2. Comparison with F-Score as Feature Selection Method

F-Score is another widely used simple and effective feature selection method, which also can measure the ability of features in discriminating different classes. For a set $X_{k}\in R^{m}$, *k* = 1, 2, ..., *n*, *l* (*l* ≥ 2) is the number of classes, *n_j_* is the number of samples belonging to class *j* (*j* = 1, 2, ..., *l*). The F-score value of the *i*-th feature is defined as

(8) $$F_{i}=\frac{\sum_{j=1}^{l}n_{j}\times {\left({\bar{X}}_{i}^{j}-{\bar{X}}_{i}\right)}^{2}}{\sum_{j=1}^{l}\sum_{k=1}^{n_{j}}{\left(X_{ki}^{j}-{\bar{X}}_{i}^{j}\right)}^{2}},$$

where, ${\bar{X}}_{i}^{j}$ and ${\bar{X}}_{i}$ are the average value of the *i*-th feature on the class *j* dataset and the whole dataset, $X_{ki}^{j}$ is the *i*-th feature of the *k*-th sample of the class *j*.

**Figure 7:**
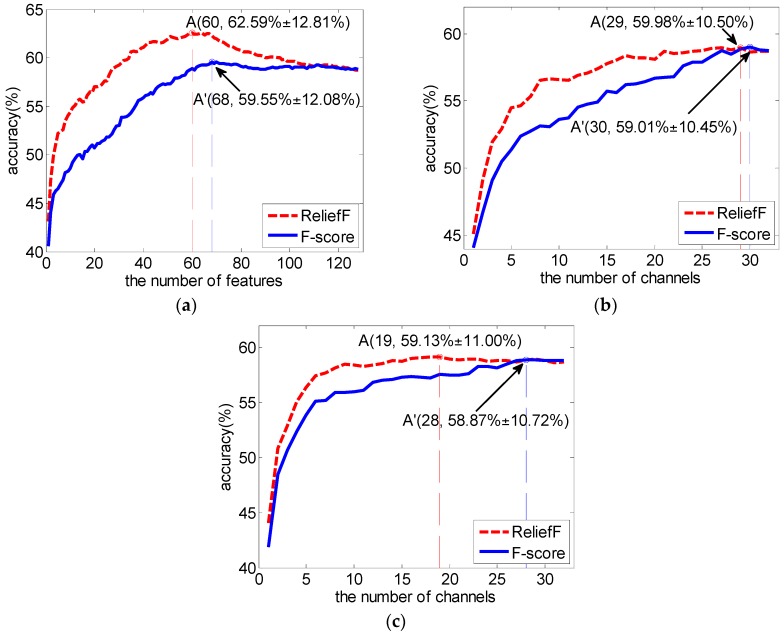
Comparison between ReliefF and F-score as a feature selection method with SVM. (**a**) a feature-selection-based channel selection method; (**b**) a channel selection method based on the channel’s weight obtained from the mean of the ReliefF or F-score weights of all features belonging to this channel; (**c**) a channel selection method based on the channel’s weight adjusted according to the channel’s contribution to classification accuracy.

We replaced ReliefF with the F-score to compute the features’ weights, and implemented the above three channel selection strategies with the new F-score weights. The results and the comparison are shown in Figure 7. It can be seen from the comparison that our RelifF-based channel selection methods gave better performance (paired *t*-test, *p* < 0.05 when the number of selected features is less than 77 in the first method (Figure 7a), and *p* < 0.05 for all 32 cases in the other two methods (Figure 7b,c).

### 3.3. Subject-Independent Channel Selection

The above results demonstrate the effectiveness of our methods, but all the experiments were performed subject-dependently. Channels were selected individually, and different among subjects. How to find common characteristics among different individuals is a natural problem. The study of selecting subject-independent channels related to emotion processing is crucial in the design of an on-line practical emotion recognition system. Furthermore, the results can help us to understand the relationship between brain regions and emotional processing.

In this study, to evaluate the importance of channels in emotion recognition subject-independently, every channel was weighted by accumulating the weight of the corresponding channels’ 16 subjects, and then ranked. For the purpose of generality, the channel’s weight for a specific subject was obtained from the mean of the ReliefF weights of all the features belonging to this channel. The subject-independent weight of the *k*th channel was computed as

(9) $$W(T_{k})=\sum_{s=1}^{SN}W(T_{sk}),$$

where, *SN* is the number of subjects (16 subjects), *W*(*T_sk_*) is the weight of the *k*th channel of the subject *s*.

**Figure 8:**
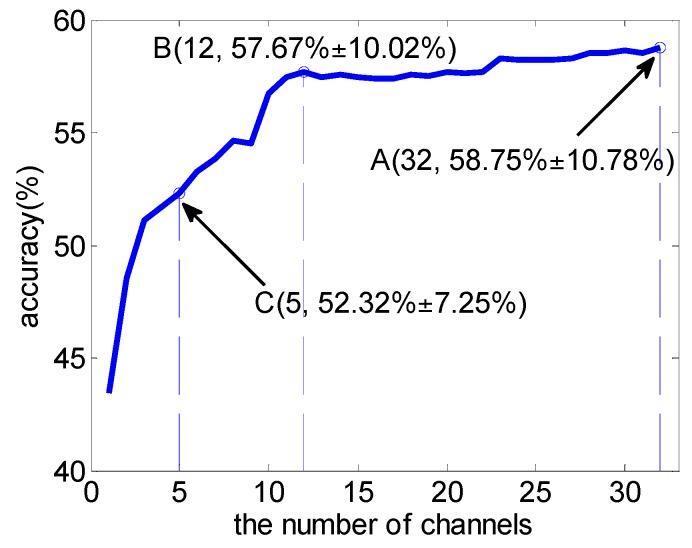
The subject-independent channel selection result.

**Figure 9:**
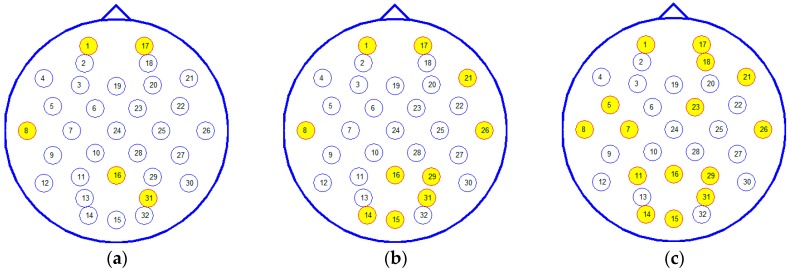
Top 5 (**a**); 10 (**b**); 15 (**c**) subject-independent channels according to a 10–20 system.

Then, the average accuracy, over a varying number of the weighted common channels in classifying four emotional states across 16 subjects, was given with classifier SVM, which is also obtained on the performance-validation dataset by five 10-fold cross-validations. The result is shown in Figure 8. Table 4 lists the top 15 common channels and Figure 9 shows the top 5, 10, 15 common channels’ locations in a 10–20 system.

Figure 8 shows that the average accuracy curve tends to be stable when the top-12 channels were selected, and the average accuracy of 57.67% ± 10.02% was obtained, compared with the accuracy of 58.75% ± 10.78% by using the top-32 channels. This result shows the possibility to design a practical subject-independent emotion recognition system using less EEG sensors. In addition, from Table 4, most top channels were located at frontal and parietal lobes and distributed symmetrically. This result was consistent with the previous report [12,18].

## 4. Discussion

Now, we will explain and discuss several important issues using the results presented above. The necessity of channel selection in EEG-based emotion recognition has been vividly verified by the results. Using any method proposed in this paper, similar trends were derived which showed that the best classification performance was achieved at a certain point, and then degraded with the increase of the channel number involved, just as we had expected. However, it should be noted that the decrease after achieving the best performance was slight, if any, so the significance of EEG channel reduction mainly lies in reducing computational complexity and facilitating user convenience, rather than improving the classification performance. Furthermore, most of the accuracy curves become almost stable before arriving at the best point. For the perspective of practical usage, the point achieving the best classification accuracy may not be the reasonable choice. We hope to find a point as a tradeoff between the classification accuracy and the number of features (channels) involved. In this respect, the MRCS and SVM-MRCS methods can give better performance in channel reduction with an acceptable loss of accuracy. It is easy to understand the better performance of the latter two methods in channel selection, because they directly evaluate a channel as a unity and use all information contained in this channel while implementing a classification task. The last method achieved the best performance among these three methods, but it should be noted that a classifier was used to adjust channels’ weights in channel selection. Although the result derived from the third method is classifier-dependent, it is still useful in practical applications where a classifier is usually determined.

From the results of subject-independent channel selection (Table 4 and Figure 9), it is shown that, with the exception of the electrodes from the frontal and parietal lobe, electrodes located in the temporal and occipital lobe also play an important role. This phenomenon is probably related with the stimuli type. In a DEAP dataset, emotional states were elicited by using music videos, including acoustic and visual stimuli which are mainly processed in temporal and occipital regions respectively. It is an interesting problem for further research.

## 5. Conclusions

We have systematically investigated ReliefF-based channel selection algorithms for EEG-based emotion recognition. Three methods were employed to quantify channels’ importance in classifying four emotional states (joy, fear, sadness and relaxation). The experiment results showed that channels involved in the classification task could be reduced dramatically with an acceptable loss of accuracy using our methods. The strategy of evaluating a channel as a unity gave better performance in channel reduction. In addition, the subject-independent channel selection problem was also investigated and achieved a good performance. The channels selected subject-independently were mostly located at the frontal and parietal lobes; they have been identified to provide discriminative information associated with emotion processing, and these channels are distributed almost symmetrically over the scalp. The results are important for the practical EEG-based emotion recognition system.

## Figures and Tables

**Table 1:** Average results (standard deviation) using top-N features and classifier SVM across 16 subjects.

Top-N Features	Average Number of Channels Involved	Average Accuracy (%)
4	3.25 (0.77)	51.04 (8.32)
8	6.56 (1.36)	53.47 (11.21)
12	9.44 (2.06)	55.13 (10.77)
16	12.13 (2.58)	55.49 (11.31)
20	14.88 (2.99)	56.99 (11.71)
24	17.50 (3.39)	57.58 (12.14)
28	19.50 (3.65)	58.93 (12.83)
32	21.44 (4.21)	59.70 (12.38)
36	23.25 (4.09)	60.69 (12.74)
40	24.50 (3.72)	61.09 (12.54)
50	27.25 (2.96)	61.86 (12.80)
60	29.63 (2.60)	62.59 (12.81)
80	31.13 (1.15)	60.76 (11.54)

**Table 2:** The average proportion of each class of features among the top-N features across 16 subjects.

Top-N Features	The Average Proportion of Each Class of Features			
	Theta	Alpha	Beta	Gamma
4	0.03	0.02	0.27	0.68
8	0.04	0.09	0.18	0.69
16	0.07	0.11	0.25	0.57
32	0.10	0.12	0.29	0.49
64	0.13	0.13	0.35	0.39
96	0.20	0.21	0.29	0.30
128	0.25	0.25	0.25	0.25

**Table 3:** The comparison of MRCS and SVM-MRCS methods using top-N channels.

Average Accuracy (Standard Deviation) Using Top-N Channels					
Top-N	MRCS	SVM-MRCS	Top-N	MRCS	SVM-MRCS
3	51.95 (8.23)	52.86 (8.41)	12	56.93 (8.95)	58.40 (10.20) *
4	52.94 (7.49)	55.08 (8.98)	13	57.06 (9.01)	58.54 (10.08) *
5	54.51 (8.30)	56.39 (8.95)	14	57.38 (9.38)	58.78 (10.27) *
6	54.66 (8.89)	57.39 (9.52) **	15	57.79 (9.34)	58.73 (10.40)
7	55.34 (8.90)	57.71 (9.53) **	16	58.05 (9.83)	58.97 (10.75)
8	56.55 (8.92)	58.16 (9.47) *	17	58.34 (9.63)	59.08 (10.92)
9	56.62 (8.98)	58.51 (10.05) *	18	58.21 (9.61)	59.10 (11.05)
10	56.56 (8.75)	58.43 (9.90) *	19	58.22 (9.80)	59.13 (11.00)
11	56.53 (8.72)	58.31 (10.05) *	20	58.09 (9.79)	58.93 (10.95)

The significant difference was tested under the same number of selected channels (paired *t*-test, * *p* < 0.05, ** *p* < 0.01).

**Table 4:** The top 15 common channels across 16 subjects.

Channel Rank	Electrode	Brain Lobe
1	Fp1	Frontal
2	T7	Temporal
3	PO4	Parietal
4	Pz	Parietal
5	Fp2	Frontal
6	F8	Frontal
7	Oz	Occipital
8	T8	Temporal
9	P4	Parietal
10	O1	Occipital
11	AF4	Frontal
12	FC5	Frontal
13	C3	Central
14	FC2	Frontal
15	P3	Parietal
